# Giant intercostal aneurysm complicated by Stanford type B acute aortic dissection in patients with type 1 neurofibromatosis

**DOI:** 10.1186/1749-8090-7-38

**Published:** 2012-04-24

**Authors:** Takeshi Uzuka, Toshiro Ito, Tetsuya Koyanagi, Toshiyuki Maeda, Masaki Tabuchi, Nobuyoshi Kawaharada, Tetsuya Higami

**Affiliations:** 1 Thoracic and Cardiovascular Surgery, Sapporo Medical University, S1W16 Chuo-ku Sapporo, 060-8543, Hokkaido, Japan

**Keywords:** Neurofibromatosis type 1, Intercostal artery aneurysm, Stanford type B acute aortic dissection, Coil embolization

## Abstract

Vascular involvement is rare in neurofibromatosis type 1 (NF1). It is often missed because it is usually asymptomatic. We report a case of a 42 years old male with neurofibromatosis type 1 who presented with left back discomfort. CT angiography revealed a massive 42 mm aneurysm of left 11th intercostal artery. After a discussion between radiologists and cardiothoracic surgeons, endovascular coil embolization was chosen to treat this patient. Percutaneous aneurysm embolization was successfully performed. However, the procedure was complicated by Stanford type B acute aortic dissection. Stanford type B acute aortic dissection was medically managed and patient remained well after discharge. Fragile vascular nature was thought to be one of the causes of this unreported complication.

## Background

Neurofibromatosis type 1 (NF1), also known as von Recklinghausen’s disease, is a well regognized genetic disorder characterized by abnormal cutaneous pigmentation (Café-au-lait spots) and multiple skin tumors. Its vascular involvement is rare but pathology such as hypertension from renal artery stenosis is well described [1]. However, large aneurysm formation of a major artery is relatively rare. Therefore, we report a case of massive intercostal aneurysm which was complicated by iatrogenic Stanford type B acute aortic dissection in a patient with NF1.

## Case presentation

### Case report

**Figure 1:**
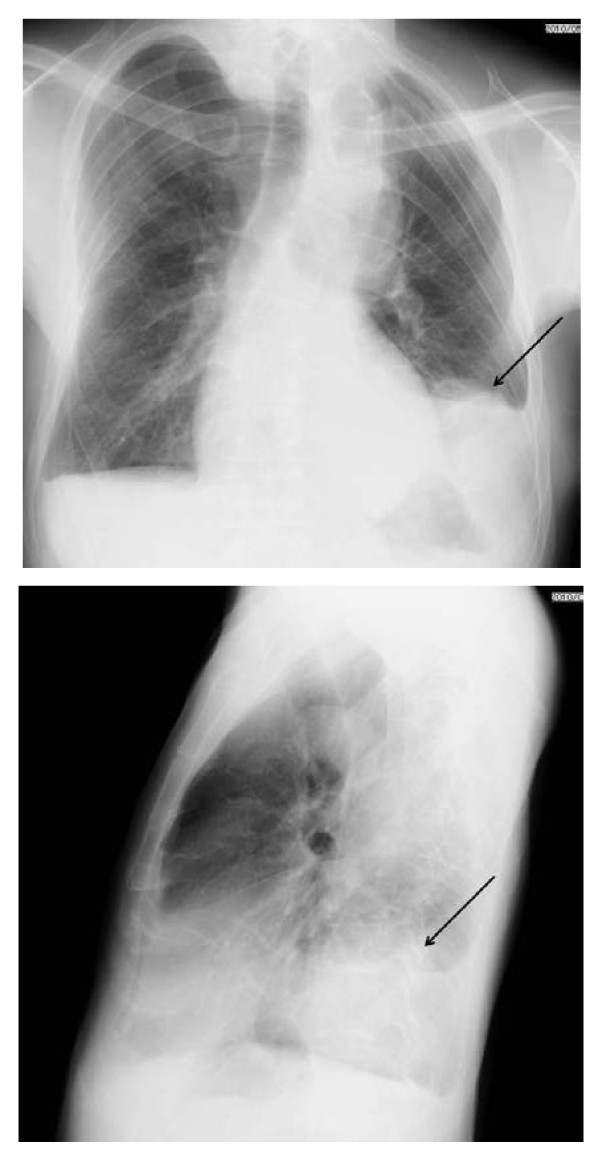
**Chest Xray.** Chest Xray shows severe scoliosis and large abnormal shadow (arrows) in left infero-posterior chest.

**Figure 2:**
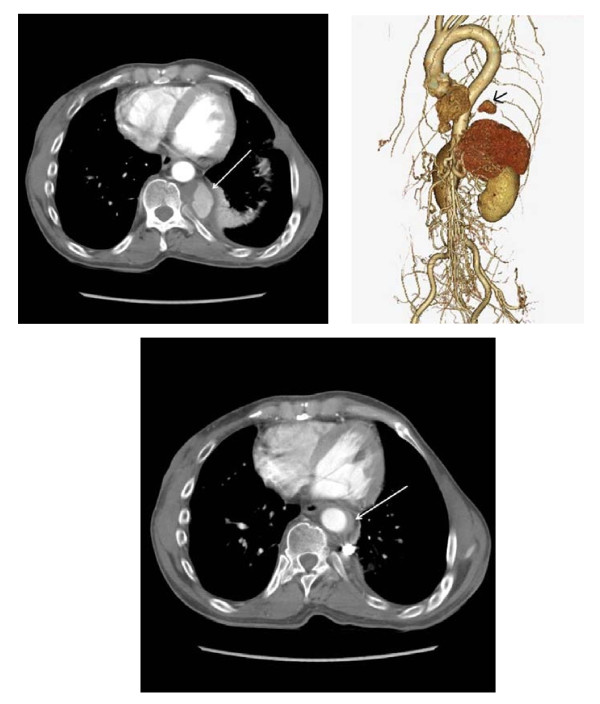
**CT.** CT showing massive intercostal aneurysm with thrombus (arrow) and mild pleural effusion (A,B). Post coil embolization CT showing early thrombosed Stanford type B acute aortic dissection (arrow).

A 42 year old male with NF1 was referred to an orthopedic surgeon for left back discomfort which didn’t resolve after 1 month. This patient was known to have a history of abdominal surgery for visceral artery aneurysm rupture. Chest Xray showed large nodular shadow in left lower chest (Figure 1). His blood test was inconclusive and tumor formation in the chest was initially suspected. However, it was found to be a 42 mm aneurysm of lt 11th intercostal artery in subsequent CT angiogram (Figure 2A, B) and patient was referred to our department for further management. After multiple discussions with radiologist, we scheduled coil embolization to treat this massive aneurysm.

Right femoral artery was punctured and used as an access to the aneurysm. Target aneurysm was identified and successfully embolized by 5 microcoils (GDC Detachable Coil, Boston Scientific). Small accessory intercostal aneurysm which could not be detected in CT angiogram was found during the procedure. It was left untouched because of its small size (<3 mm). Besides, early thrombosed Stanford type B acute aortic dissection had developed in adjacent descending aorta with new sharp back pain during the contrast injection after the coil embolizatin (Figure 2C). Patient received intensive medical therapy for blood pressure control and thrombosed false lumen in descending aorta regressed eventually. Patient was discharged to home on day 14 and remained well.

## Discussion

Neurofibromatosis type 1 (Von Recklinghausen’s disease) is a well recognized autosomal dominant disorder associated with chromosome 17q11.2 [2]. It affects one in 3000 people and involves any organ in the body primarily connective tissue and nerve tissue and others [3,4]. Diagnosis has been usually made by macroscopic features (café au-lait spot and multiple skin neurofibromas) and family background as it was in this patient. Majority of the patients are asymptomatic but formation of visceral neurofibroma and higher propensity of malignant transformation in any organ lead to increased morbidity and mortality of this disease. Vascular involvement is not common but arterial stenosis due to intimal or medial dysplasia is a well known and renovascular hypertension due to renal artery stenosis is a well documented vascular complication [1]. Some cases of aneurysm formation have also been reported as a result of friable vascular tissue [4-8]. The aneurysm can be formed in any vessels in the body such as renal, intercostal, carotid-vertebral or intracranial arteries [1,5,7-10]. Signs and symptoms depend on the size and location of the aneurysm and may include ischemic, compression or rupture symptom from the involved artery.

Two treatment options for intercostal aneurysm have been reported; surgery and percutaneous coil embolization. Although surgical resection used to be the only option, with the advance of current interventional radiology, percutaneous coil embolization is becoming a good option to treat this type of disease especially for stable patient [9,11]. In our case, radiologists in our institution were concerned about the fragile nature of vessels in NF1 patient [11,12], and initially hesitated to do this procedure. However, we thought open surgery such as clipping or excision of aneurysm is rather invasive and also can be difficult because of the fragile characteristics of the artery in NF1 [8,11,13]. It may bring unexpected disastrous complication like paraplegia from postoperative hematoma compression [8] especially when aneurysm is close to the spine as it was in this case. Therefore, we thought percutaneous coil embolization was safer option. And I believe it brought acceptable outcome in this case, although it was complicated by the Stanford type B acute aortic dissection which was managed successfully with conservative medical therapy.

Apart from a report regarding spontaneous aortic dissection [14], acute aortic dissection as a complication of percutaneous coil embolization in NF1 patient has not been described before. We surmise underlying fragile vascular condition is related to this possible but potentially fatal complication. The fact that dissection did not localize in intercostal artery may imply that all vessels in the body may hold this fragile vascular nature. Therefore, special attention needs to be taken during/after the procedure to prevent unnecessary vascular complication.

This patient had a history of ruptured visceral aneurysm. Although we could not find the result of histopathological test from this first surgery, we suspect this was also related to NF1 because NF1 can affect any part of the body and multiple aneurysm formation is also possible vascular pathology. We accidentally found another small (<0.5 cm) intercostal aneurysm during the coil embolization. We decided not to treat this accessory aneurysm together because of its small size. However, treatment indication is unclear in this rare condition and it may hold higher probability of progression/rupture due to the fragile vascular tissue [11]. Therefore, careful follow up with regular radiographic assessment is necessary for this patient.

## Conclusions

We have experienced a rare case of huge aneurysm of left 11th intercostal artery in a patient with NF1. Percutaneous coil embolization was successfully performed. However, this case was complicated with Stanford type B acute aortic dissection during the procedure. Vascular tissue is thought to be fragile in this type of disease and special attention is required not only during the procedure but also at the clinic.

### Consent

Patient has given a consent for publication of this case report and any accompanying images.

## Abbreviations

NF1, Nerofibromatosis type 1; CT, Computed Tomography; GDC, Guglielmi Detachable Coils.

## Competing interest

The authors declare that they have no competing of interest.

## Authors’ contribution

IT drafted the manuscript and TH supervised entire treatment. IT and TK and TM and MT and NK participated the treatment. All authors read and approved the final manuscript.
